# Currently Used Methods to Evaluate the Efficacy of Therapeutic Drugs and Kidney Safety

**DOI:** 10.3390/biom13111581

**Published:** 2023-10-26

**Authors:** Hung-Jin Huang, Chu-Lin Chou, Tin Tin Sandar, Wen-Chih Liu, Hsiu-Chien Yang, Yen-Chung Lin, Cai-Mei Zheng, Hui-Wen Chiu

**Affiliations:** 1 Division of Nephrology, Department of Internal Medicine, School of Medicine, College of Medicine, Taipei Medical University, Taipei 110, Taiwanchulin.chou@tmu.edu.tw (C.-L.C.);; 2 Division of Nephrology, Department of Internal Medicine, Hsin Kuo Min Hospital, Taipei Medical University, Taoyuan City 320, Taiwan; 3 TMU Research Center of Urology and Kidney, Taipei Medical University, Taipei 110, Taiwan; 4 Cancer Epidemiology Unit, Oxford Population Health, University of Oxford, Oxford OX3 7LF, UK; 5 Department of Biology and Anatomy, National Defense Medical Center, Taipei 114, Taiwan; 6 Section of Nephrology, Department of Medicine, Antai Medical Care Corporation Antai Tian-Sheng Memorial Hospital, Pingtung 928, Taiwan; 7 Division of Nephrology, Department of Internal Medicine, Zuoying Branch of Kaohsiung Armed Forces General Hospital, Kaohsiung 813, Taiwan; 8 Division of Nephrology, Department of Medicine, Tri-Service General Hospital, National Defense Medical Center, Taipei 114, Taiwan; 9 Division of Nephrology, Department of Internal Medicine, Taipei Medical University Hospital, Taipei 110, Taiwan; 10 Division of Nephrology, Department of Internal Medicine, Shuang Ho Hospital, Taipei Medical University, New Taipei City 235, Taiwan; 11 Graduate Institute of Clinical Medicine, College of Medicine, Taipei Medical University, Taipei 110, Taiwan; 12 Department of Medical Research, Shuang Ho Hospital, Taipei Medical University, New Taipei City 235, Taiwan; 13 Ph.D. Program in Drug Discovery and Development Industry, College of Pharmacy, Taipei Medical University, Taipei 110, Taiwan

**Keywords:** biomarkers, kidney disease models, alternative methods, acute kidney injury, chronic kidney disease

## Abstract

Kidney diseases with kidney failure or damage, such as chronic kidney disease (CKD) and acute kidney injury (AKI), are common clinical problems worldwide and have rapidly increased in prevalence, affecting millions of people in recent decades. A series of novel diagnostic or predictive biomarkers have been discovered over the past decade, enhancing the investigation of renal dysfunction in preclinical studies and clinical risk assessment for humans. Since multiple causes lead to renal failure, animal studies have been extensively used to identify specific disease biomarkers for understanding the potential targets and nephropathy events in therapeutic insights into disease progression. Mice are the most commonly used model to investigate the mechanism of human nephropathy, and the current alternative methods, including in vitro and in silico models, can offer quicker, cheaper, and more effective methods to avoid or reduce the unethical procedures of animal usage. This review provides modern approaches, including animal and nonanimal assays, that can be applied to study chronic nonclinical safety. These specific situations could be utilized in nonclinical or clinical drug development to provide information on kidney disease.

## 1. Introduction

Kidney-related diseases, such as acute kidney injury (AKI), diabetic kidney disease (DKD), chronic kidney disease (CKD), and end-stage renal disease (ESRD), continue to remain a considerable problem in the clinic. For preclinical safety assessment, nonclinical biomarkers are used to determine how well the body responds through biochemical changes in both nonclinical species and humans to assess the safety and efficacy of novel compounds [1]. Traditionally, drug developers have used biochemical markers to detect renal injury. However, there is room for innovation in discovering novel markers of kidney disease in drug development. The research of uncovered new biomarkers has two main scientific styles: hypothesis-based and discovery-based investigations [2]. The candidate biomarkers identified by the hypothesis-based approach are based on the knowledge of disease processes with the product of ever-increasing mechanistic understanding [3]. For instance, detecting glycosylated hemoglobin with a sustained elevation of blood glucose levels can be a biomarker for diagnosing diabetes mellitus. The discovery-based method focuses on monitoring the changes in the abundance of molecular species associated with the disease of interest. An example of a discovery-based study is the search for the BRCA1 gene in breast and ovarian cancer, which was frequently lost in the region of chromosome 17q21 in breast cancer [4].

Traditionally, the diagnosis or prognosis of renal disease based on biomarkers is essential for developing and evaluating potential treatments. The prognostic biomarkers determine the relationship between the disease and clinical outcome in the absence of medication and therapy, while the predictive biomarkers measure whether a patient is most likely to benefit from a particular treatment [5]. Biomarkers can be used to measure quantitative parameters, such as genes [6], proteins [7], microRNAs [8], lipids [9], and metabolites in serum [10], to examine whether the kidney function of patients is healthy or abnormal on urinalysis. Therefore, they are essential to evaluate pharmacological responses and the pathogenesis of diseases for the therapeutic intervention of kidney disorders. However, blood urea nitrogen (BUN) and serum creatinine (SCr) are not very accurate and specific markers of kidney function in acute coronary syndrome [11]. For instance, the BUN and SCr are not accurate renal function indexes due to the abnormal BUN/SCr ratio observed in patients with heart failure [12]. The levels of the serum biomarkers mentioned above and urine biomarkers among patients with liver failure were affected by numerous factors, including ingestion of creatinine, excretion of drugs, protein intake, sex, and age [13]. Loss of muscle mass will lead to overestimating the glomerular filtration rate (GFR). Thus, using SCr to assess kidney function accurately is prone to error in steady-state situations [14]. Therefore, this review summarized the potential biomarkers to improve renal function deterioration and detection accuracy in the early stage of kidney damage.

Much fundamental research has made unprecedented progress in detecting novel kidney biomarkers [15,16]. Early diagnostic methods benefit from choosing the most appropriate preclinical drug and the mechanistic understanding of human nephropathy. So far, studies on blood and urine biomarkers in renal biopsy of animals are widely used strategies to understand the mechanisms of kidney-related disease and to develop successful treatments. Nevertheless, considering the ethical treatment of animals, the 3Rs principles of replacement, reduction, and refinement were developed to avoid unethical research more than 50 years ago [17]. Various practical alternative methodologies have been created for assessing the possible harmful substances of chemicals. The 3Rs offer a method for reducing animal usage and suffering in studies while maintaining the standard of scientific work being done logically and step-by-step. Today, alternative methods are important in reducing the number of experimental animals for hazard identification and safety assessments. Advanced alternative strategies, including in vitro and in silico methods, are also used for kidney safety assessment or prediction during drug development [18]. To study the multiple causes of kidney failure, many mammalian model systems have been used to investigate the mechanisms of renal injury progression and identify potential targets for early clinical trials of lead drug discovery [19,20,21].

However, recent studies have indicated that animal models do not precisely simulate human diseases or biological processes [22,23]. In addition, alternative methods have become essential for animal experimental replacement to provide more ethical and humane research approaches [24,25]. Advanced alternative methods, such as in vitro cell culture, human organoids, and in silico methods, provide unique opportunities for the safety assessment of drug candidates and for studying human disease to overcome these limitations [26,27,28]. For example, integrated testing strategies using computer models and some instruments (e.g., mass spectrometry, functional and pharmacological magnetic resonance imaging, and flow cytometry) have facilitated measurable progress to produce valuable data [29]. The advantages of the above technologies lead to a reduction in the number of animals and help to achieve the goals of the 3Rs in research. Hence, these methods may provide a powerful strategy for developing appropriate drugs and identifying new therapeutic approaches. This review presents a series of modern clinical and nonclinical research strategies and summarizes the valuable tools of current assessments, including models of kidney disease and alternative methods.

## 2. Protein Biomarkers of Kidney Safety to Predict and Diagnose Kidney Disease in Preclinical and Clinical Studies

The term biomarker is short for “biological marker”, also called molecular marker, and is a signature molecule found in body fluids, blood, and tissues in normal conditions or disease [30]. Generally, biomarkers indicate the physiological status and may be used as analytic or measurable disease features to monitor the body’s responses [31,32]. Biomarkers also play an important role in developing medical devices and lead compounds during drug development [33], providing valuable data after measuring the responses of pharmacodynamics, pathogenic processes, and biological processes after treatment with medical interventions [34]. However, there is considerable misunderstanding about the biomarkers’ definitions and ability to serve as predictive, diagnostic, and prognostic markers. Biomarkers can be categorized into seven kinds of detection: predictive [35], susceptibility [36], diagnostic [37], prognostic [38], monitoring [39], pharmacodynamic [40], and safety [41]. Moreover, these approaches have the potential to provide a powerful tool for the development of new therapeutic strategies and for monitoring the benefits of administered drugs. Predictive biomarkers can often measure observational data in response to therapeutic interventions of interest before treatment and provide information in clinical practice for preventing kidney failure [42]. The important protein biomarkers of kidney safety for assessing kidney disease status are discussed in the following subsections.

### 2.1. Protein Biomarkers for the Prediction, Diagnosis, and Prognosis of Kidney Injury

**Figure 1:**
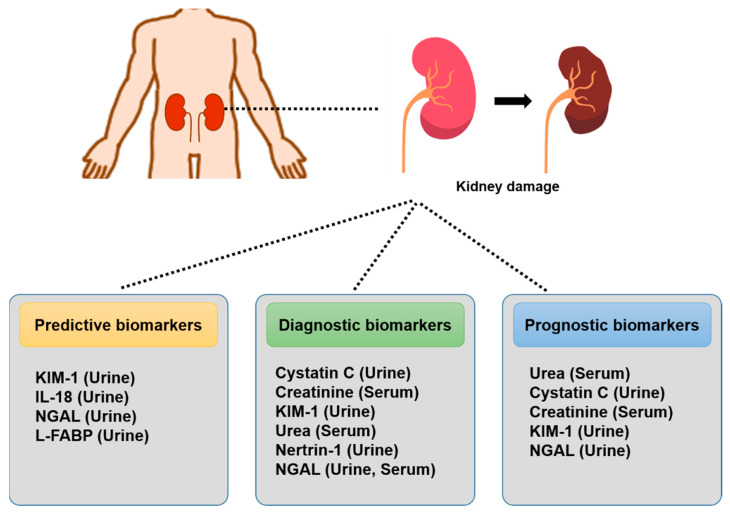
Biomarkers of kidney injury: the traditionally used markers in blood and urine are predictive, diagnostic, and prognostic characteristics of the different processes of kidney injury. Serum and urinary biomarkers of kidney disease facilitate earlier detection and prevention to establish effective therapies.

The traditionally used endogenous marker can be predictive, diagnostic, and prognostic in the different stages of kidney failure (Figure 1). The predictive biomarkers approved by the FDA and EMEA for detecting kidney damage [43] include the urine biomarkers of kidney injury molecule-1 (KIM-1) [44], urinary neutrophil gelatinase-associated lipocalin (NGAL) [45], interleukin-18 (IL-18) [46], and liver fatty acid-binding protein (L-FABP) [47]. Prognostic biomarkers can be a clinical or biological characteristic that provides information from patients with high risk for associated outcomes irrespective of therapy. The prognostic biomarkers of kidney injury include urine biomarkers (KIM-1, cystatin C, netrin-1, and NGAL) and blood biomarkers (creatinine, urea, and NGAL). Prognostic biomarkers can be used to predict the course of disease from observational data and help identify the outcomes of patients with medical conditions or interventions. The prognostic biomarkers associated with kidney disease included urea and creatinine in blood and KIM-1, NGAL, and cystatin C in urine. Urine and serum contain promising biomarkers that can aid in the early detection of kidney injury and identify the mechanism of injury. Thus, they are valuable tools for diagnosis at an early stage to minimize the severity of injury.

### 2.2. Biomarkers of Inflammation

The inflammatory response and its activation pathways in kidney cells can produce early responses to kidney injury, and various molecules, such as growth factors, chemokines, adhesion molecules, proinflammatory cytokines, and molecular signatures, can be triggered and involved in the inflammatory process. Regarding the biomarkers of kidney inflammation, several previous studies illustrated that the adhesion molecules intercellular adhesion molecule-1 (ICAM-1) [48] and vascular cell adhesion molecule-1 (VCAM-1) [49] and the inflammatory cytokines interleukins (1, 6, 8, 17, 18, and 19) [50,51,52,53], tumor necrosis factor receptors (TNFR1 and TNFR2) [54], nuclear factor kappa B (NF-κB) [55], activator protein 1 (AP-1) [56], C-reactive protein (CRP) [57], and monocyte chemoattractant protein-1 (MCP-1) [58] are the most commonly used mediators to detect inflammation in clinical or nonclinical research.

TNF receptors belong to a protein superfamily of cytokine receptors, which contain glycoproteins with a single-pass transmembrane domain to transmit signals for downstream signaling. Inflammatory responses are mediated by the binding of TNF-α to TNF receptors and contribute to apoptosis through the activation of NF-κB and AP-1. Recently, TNFR1 and TNFR2, biomarkers of low-grade inflammation, are important indicators for kidney disease diagnosis [59]. Additionally, the inflammatory cytokine TNF-α is the most extensively studied molecule. For instance, patients with renal failure generally have higher expression levels of TNF-α than healthy patients [60]. During the progression of the inflammatory response by kidney injury, patients with albuminuria have higher levels of TNF-α than patients without albuminuria. Another previous study demonstrated that the TNF receptor TNFR2 protein binding to TNF-α was positively associated with albuminuria and tissue injury in the progression of diabetic nephropathy [61]. Therefore, TNF-α can serve as an important indicator in the progression of kidney injury and has been regarded as an attractive target of inflammatory cytokines in developing novel therapeutic agents for kidney failure. Regarding CKD, early studies have also illustrated that increased levels of circulating TNFRs are strongly related to the progression of kidney damage caused by diabetes, stage 3 CKD, and end-stage kidney disease [62].

### 2.3. Biomarkers of Nephrotoxicity

Traditionally, safety biomarkers can provide crucial technical assistance in risk assessment and can be broadly used to monitor kidney safety after exposure to a medical product. Hence, the use of safety biomarkers can inform the extent of toxicity and assist in the early detection of kidney injury to predict the clinical nephrotoxicity risk during the progression of drug treatment. In early drug development, the primary challenge is accurately assessing toxicity risk to differentiate compounds in clinical trials [63]. Traditional nephrotoxicity biomarkers, including BUN and serum creatinine, have poor timing accuracy and low sensitivity for diagnosis. Therefore, precise or sensitive safety biomarkers are needed for a more specific diagnosis of nephrotoxicity for risk minimization. The outcome may help identify kidney failure’s severity after intervention in nephrotoxicity signaling. The promising prognostic biomarkers in different kidney units for monitoring nephrotoxic drugs are described in Table 1. For instance, the proinflammatory cytokines or chemokines released by leukocytes, including IL-6, IL-18, IL-10, MCP-1, IFNγ, TGF-β, and CXCL1, were the target of preclinical studies or drug development in proximal tubular epithelial cells for the early diagnosis of AKI [64]. Some studies have demonstrated that IL-18 is a crucial mediator of acute injury in the renal parenchyma and tubular cells. These biomarkers mentioned above are more specific and sensitive for detecting AKI at an earlier stage of kidney injury, and a rapid diagnosis can be applied to identify patients with a high risk of AKI. Approximately one hundred traditional biomarkers were discovered from previous reports to evaluate safety risk. No unrivaled biomarker is available in routine clinical practice [65].

## 3. Currently Used Experimental Models to Assess the Drug Mechanism in Kidney Therapy

Experimental animal models have been widely used to investigate nephropathy events, study the underlying mechanisms of renal disease, discover specific biomarkers, and identify potential targets for kidney therapy [65,66]. AKI can be regarded as an irreversible renal function that increases the risk of CKD’s development. In this section, we focus on mouse and rat models that mimic human AKI and CKD to study the efficiency and safety of interventions and treatments of interest. Current models of AKI and CKD can be induced by endogenous toxins (sepsis), ureteral obstruction, ischemia–reperfusion (IR), treatment with chemicals (cisplatin, folic acid, etc.), unilateral nephrectomy, and hypertensive nephropathy. The above critical information on the current various animal models is summarized in Table 2. Different murine models of mice and rats are relatively inexpensive and easily maintained. However, the recently available models, including their model techniques, the types of models, and modeling time, are not mentioned clearly. Here, we will discuss the platform of AKI and CKD models, including their engineered method, surgical operations, model time courses, disease induction of toxins, and the dose ranges for treatment.

### 3.1. Sepsis-Induced Models

Generally, sepsis-associated AKI can be considered severely inflammatory and has become a frequently studied model in rats and mice [79]. The major models used for AKI induction have two types of strategies: (1) injection of bacteria or endogenous toxins to establish a lipopolysaccharide (LPS) mouse model that triggers stronger immunity [80], and (2) two needle punctures into the abdominal cavity to create the symptoms of bacterial peritonitis as a cecal ligation and puncture (CLP) model [81]. The commonly used experimental drug dosage is 10–15 mg/kg LPS [82]. The Toll-like receptor 4 (TLR-4) of immune cells is a specific receptor that can interact with LPS to secrete inflammatory cytokines such as IL-1, IL-6, and TNF-α, leading to sepsis and inflammation at 72–96 h [83]. However, it is difficult to regulate the severity of sepsis in mice, and it is not easy to develop reproducible AKI in CLP models [84].

### 3.2. Chemical-Induced AKI

Endogenous toxins or poisons are used to induce acute renal failure and play an essential role in developing cardiac surgery-associated AKI [85]. A chemotherapy agent, cisplatin, is widely used in solid tumor therapy [86]. However, high doses of cisplatin can cause nephrotoxicity in humans, leading to AKI or CKD. In particular, the adverse effects of cisplatin are injury to proximal tubules and cell apoptosis or necrosis through oxidative stress, calcium overload, and the inflammatory response. Cell death caused by cisplatin reduces the GFR value [87] and increases vascular resistance [88]. In addition, i.p. injection of aristolochic acid can induce AKI and CKD, which cause proximal tubular cell necrosis and result in kidney fibrosis and oxidative stress. Several studies have employed aristolochic acid-induced kidney failure to explore new therapeutic targets [89].

## 4. In Vitro and In Silico Alternative Models to Animal Tests for Drug Safety Assessment

**Figure 2:**
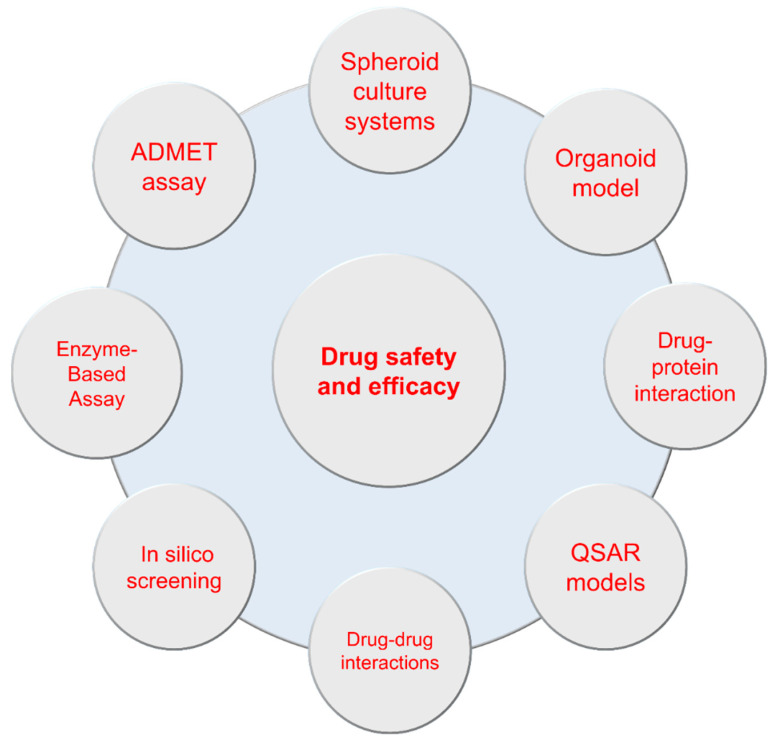
Schematic representation of identification techniques, protein assays, bioinformatics techniques, and molecular dynamics simulations for drug safety and efficacy assays.

Traditionally, millions of mammals, including mice and rats, are widely used to identify hazards and safely assess the tested compounds. However, the 3Rs principle defined by W.M.S. Russell (1925–2006) and R.L. Burch (1926–1996) has become essential for minimizing animal use in evaluating potential toxic chemicals over the past years. Several alternative methods, such as in vitro and in silico models, can replace and reduce the number of living animals for modern toxicologists, offering substantial cost savings and increasing the effectiveness of new drug discovery. The advantage of in vitro and in silico models is that they allow us to test drug efficacy and toxicity without animal experiments. Therefore, in vitro models offer new opportunities to model in vivo conditions. To develop nonanimal model alternative methods to support the criteria of consciousness in animals, including replacing, reducing, and refining, the currently used alternative approaches are summarized in Figure 2. The following section discusses potential techniques for the replacement of animals to explore the effects of and responses to drugs and identify genetic pathways involved in the development of drugs.

### 4.1. In Vitro Renal Models

Traditional in vitro assays and two-dimensional (2D) cell culture systems are widely used in vitro techniques for exploring the mechanisms of diseases, nonclinical testing, drug discovery, and drug risk assessments. The advantages of 2D cultures are low maintenance cost and simple reproducibility [90], and the cells grow as a monolayer in a flat petri dish via adherent 2D cultures to attach the surface [91]. Therefore, most research utilizes 2D cultures to study lead compounds’ pharmacology, efficacy, and safety in biological systems [92]. Over the past decades, the pharmaceutical industry has also utilized 2D cultures to evaluate the properties of pharmacokinetics, such as absorption, distribution, metabolism, and excretion (ADME), and to perform drug risk assessments of newly discovered chemicals in drug development [93]. The proximal tubule cell is a vital nephron segment and is usually regarded as a research target in the nephrotoxic injury study [94]. Developing proximal tubule cell lines from human cells enables researchers to model common types of nephropathies. For instance, in vitro kidney cell models have potentially mimicked the change from normal kidney tissue to early pathologic CKD conditions [95]. A single cell type in a 3D environment in vitro also enables the use of several recently established tissue engineering (TE) techniques as kidney disease models, such as polycystic kidney disease [96], renal cell carcinoma [97], nephronophthisis [98], drug-induced nephrotoxicity [99], and kidney fibrosis [100]. Therefore, much research has focused chiefly on proximal tubule cells to investigate nephrotoxic compounds for safety assessment [101,102]. However, the proximal tubules reabsorb glucose, electrolytes, and albumin that can transport drugs and secrete xenobiotics into the filtrate to disrupt nephrotoxic studies. In addition, the microvasculature in renal cells is also susceptible to drug-induced injury and sensitive to phosphorylation, which can cause inflammation with consequences on tubule function [103].

### 4.2. Organoid Models of Kidney Diseases

To date, in vitro 2D systems for studying nephrotoxicity are still poorly predictive of toxicity in animals or humans [104]. The major disadvantages of 2D culture are that it does not represent cell–cell and cell–ECM interactions [105]. Thus, there is a need to find better alternative models to mimic natural cell growth and the reality of diseased tissue, such as 3D culture systems. The 3D models appear to better represent animal tissue outside the living species than 2D cell culture models [106]. Undoubtedly, the ideal environment in disease modeling in drug development is 3D cell culture systems instead of traditional monolayer cell cultures and animal models, and they have been used to understand human biology and disease modeling during the past decade [107]. However, the above experimental models still have challenges in mimicking the physiological environment of organs due to the low capacity to fully recapitulate the organization of tissue under in vivo environments [108]. For instance, the vascularization of organoid culture systems in in vitro models can overcome the limitations of mimicking organoid-vasculature interactions to avoid necrotic core formation [109]. Vascularized kidney organoids have recently been derived from human pluripotent stem cells (hPSCs) to mimic in vivo nephrogenesis [110]. The disease modeling applications using hPSC-derived kidney organoids can be applied to study the progression of MUC1 kidney disease [111], ITF40 Nephronophthisis [112], polycystic kidney disease [113], and podocalyxin deficiency [114]. In addition, the kidney organoids also mimic signal cascades and growth factor stimulation. They consist of segmented nephrons and stromal cells and have critical features of the kidney organ [115]. iPSC-derived kidney organoids have been employed in various research fields, including toxicity studies, cell therapy, disease modeling, and drug development [116].

## 5. In Silico Models for Predicting Drug Safety and Efficacy

**Figure 3:**
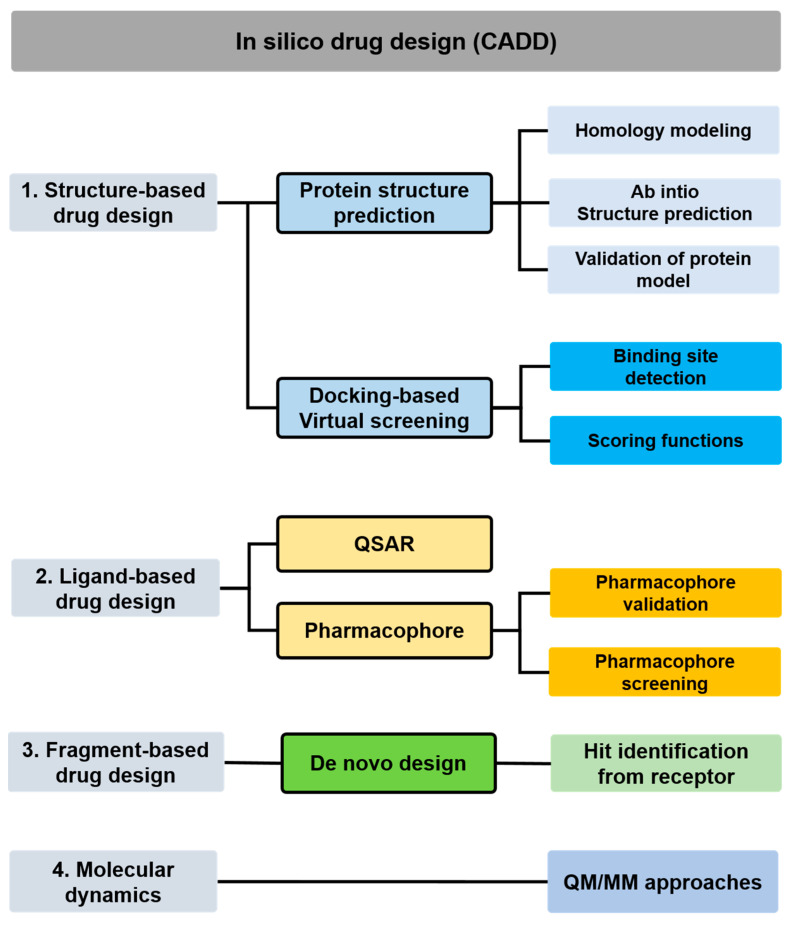
Flowchart of various CADD methods used in drug discovery (abbreviations: CADD: computer-aided drug design; QM: quantum mechanical; MM: molecular mechanical; QSAR: quantitative structure-activity relationship).

The in silico methods for drug design have numerous advantages for modeling pharmacologic or physiologic processes throughout the pharmaceutical industry. For instance, computer-aided drug design (CADD) has become essential to modern drug discovery in different research environments to accelerate therapeutic drug development (Figure 3). One in silico strategy is based on physical knowledge to perform high-throughput virtual screening (HTVS) from existing chemical libraries with millions of small chemicals, including the ZINC database [117], DrugBank [118], and e-Drug3D [119] database, for the identification of potential hits and repurposing of approved molecules for the target of the disease.

### 5.1. Structure-Based In Silico Methods

Docking-based virtual screening uses computational simulation to evaluate chemical-biomolecule interactions based on 3D structure information. To further study the binding modes of small molecules or macromolecules at the atomic level, molecular dynamics (MD) simulation techniques provide more information or significant complementarity by calculating the potential energy of a system with given positions in space. For instance, quantum mechanics/molecular mechanics (QM/MM) MD simulations combine the accuracy of ab initio QM calculations and speed simulation approaches to study every atom in a protein molecular system and solution. This approach is favorable in investigating features of protein–ligand, protein–protein, protein–nucleic acid, and protein–peptide complexes. Based on a broad model of the physics driving interatomic interactions, MD simulations forecast how each atom in a protein or other molecular system will move over time. The MD simulations can obtain a wide variety of critical biomolecular processes, such as conformational change, ligand interaction, and protein folding, revealing the positions of all the atoms at femtosecond temporal resolution. Importantly, these simulations can also forecast how biomolecules react to alterations like mutations, phosphorylation, protonation, or the addition or removal of ligands at the atomic level. A wide range of experimental structural biology techniques, such as x-ray crystallography [120], cryo-electron microscopy (cryo-EM) [121], nuclear magnetic resonance (NMR) [122], electron paramagnetic resonance (EPR) [123], and fluorescence resonance energy transfer (FRET) [124] are frequently utilized in conjunction with MD simulations. To study the treatments for kidney failure, MD simulations can be applied to investigate several innovative drugs for treating kidney diseases. For instance, studying the mechanical interaction of a ligand on the dynamics of the genetic risk of apolipoprotein L1 (APOL1) enables the discovery of potential inhibitors for CKD disease [125].

### 5.2. Ligand-Based In Silico Methods

This strategy uses the target’s 3D structure, which is available to determine the nature of the target-ligand interaction and the ligand’s structural requirements. The statistical methods used for ligand-based drug design, such as quantitative structure-activity relationship (QSAR) and pharmacophore, construct relationship models between biological activity and structural information using mathematical algorithms, including linear or nonlinear methods for model generation. The QSAR and pharmacophore approach can be widely utilized in scientific predictions and prevention assessments, such as toxicology assessment, risk prediction, and regulatory decisions. Thus, the ligand-based model can replace animal tests in predicting hazardous substances and analyzing the potentially toxic effects of experimental materials or chemicals in current years. Due to the lack of an experimental structure, the known ligand molecules that bind to the drug target are investigated to determine which of the ligands’ structural and physico-chemical characteristics correspond to the required pharmacological activity of those ligands [126]. In addition to recognized ligand molecules, ligand-based techniques may use natural products or analogs of substrates that interact with the target molecule to produce the desired pharmacological outcome [127]. To study kidney toxicity, the equations of QSAR modeling allow researchers to identify and understand their inhibitory effects related to critical structural features on the renal cells [128]. As an alternative, structure-based approaches, including molecular docking or in silico chemical modification, are typically used for lead optimization when the drug target has a 3D structure [129].

## 6. Conclusions

Several validated and prevalidated methods can be used as partial or complete alternatives to animal experiments to study the efficacy and toxicity of lead compounds. However, animal ethics is an important issue during the process of hazard identification and assessment of potential biomarkers during the process of kidney injury. The concept of the 3Rs principles facilitates various alternative methods that require investigators to replace traditional animal tests. Several alternative methods, including cell-based methods and computational models, offer an opportunity to reduce the number of experimental animals needed. Despite the reasonable new insights into kidney disease obtained from existing models, many do not fully reproduce kidney disease. Hence, improving kidney disease models is still an important issue. This review has provided a brief overview of the currently used animal models and alternative assessment methods that possibly parallel human kidney disease to offer valuable insights into the pathogenesis of this disease.

## Figures and Tables

**Table 1:** List of promising prognostic biomarkers of nephrotoxicity induced by drug with cytotoxicity.

The Functional Unit of the Kidney	Biomarkers	Nephrotoxic Drug
Proximal tubule	Interferons, Interleukins, TNF, CSFs, KIM-1, NGAL, Clusterin, Osteopontin, NAG, Beta-2 microglobulin, Albumin	Aminoglycoside, Antibiotics, Amphotericin B, Adefovir, Cyclosporine, Cisplatin, Foscarnet Contrast stain, Cocaine, Heroin, Methadone Methamphetamine Gentamicin, Vancomycin
Distal tubule	Serum cystatin C, NGAL, Clusterin, Osteopontin	Aminoglycosides, Amphoterin B, Radiocontrast dye, Methotrexate, Vancomycin
Glomerulus	Interferons, Interleukins, TNF, CSFs, Type IV collagen	ACE inhibitor, ARB, NSAIDs, Mitomycin-C, Antiplatelet, Cyclosporin, Quinone

ARB: angiotensin II receptor blockers, NSAIDs: nonsteroidal anti-inflammatory drugs, TNF: tumor necrosis factor, CSFs: colony-stimulating factors, NAG: N-Acetyl-Beta-D-Glucosaminidase, NGAL: Neutrophil Gelatinase-Associated Lipocalin, KIM-1: Kidney Injury Molecule-1, ACE: angiotensin-converting enzyme.

**Table 2:** Summary of major used experimental AKI and CKD models via traditional animal modeling approaches.

Pathology	Experimental Models	Experimental Strategy	References
Sepsis-induced AKI	Sepsis	Sepsis induced by intraperitoneal injection of 10 mg/kg LPS	[67]
Obstructive AKI	Unilateral ureteric obstruction	1–2 weeks of induction for renal fibrosis	[68]
	Ischemia-reperfusion injury	Bilateral renal ischemia for 30 min and then treatment with reperfusion for 24 h	[69]
Chemical induced AKI	Cisplatin	20 mg/kg of cisplatin within 72 h	[70]
	Folic acid	A single dose of 250 mg/kg Folic acid injection for AKI induction	[71]
	Glycerol	24 h of water deprivation and a single injection of 3–30 mg/kg 25% glycerol	[72]
	Gentamicin	Dose range 30–200 mg/kg for 4–8 days	[73]
	Warfarin	5/6 nephrectomy rats for 3 weeks and then treated with warfarin for 8 days	[74]
CKD caused by mass reduction	5/6 nephrectomy (rats)	Renal mass reduction by one-sided nephrectomy after removal of the right kidney 1 week	[75]
CKD induced by hypertension	Angiotensin II infusion models	Induction of fibrosis by the increase in the circulating level of angiotensin II	[76]
Diabetic nephropathy	Streptozotocin mice/rats	A single dose of 200 mg/kg Streptozotocin is directly toxic to pancreatic beta-cells	[77]
Membranous nephropathy	Heymann nephritis rats	Anti- fraction 1A (Fx1A) antiserum in Sprague-Dawley rats	[78]

## Data Availability

Not applicable.
